# The Effect of Lifestyle Food on Chronic Diseases: A Comparison between Vegetarians and Non-Vegetarians in Jordan

**DOI:** 10.5539/gjhs.v5n1p65

**Published:** 2012-11-04

**Authors:** Nizar Issa Alrabadi

**Affiliations:** 1 Faculty of Agricultural Sciences, Jarash University, Jarash, Jordan

**Keywords:** vegetarian, non-vegetarian, chronic diseases, diabetes, hypertension, obesity

## Abstract

Foods do certainly play an important role in human health. This cross sectional study investigated the effect of lifestyle food on chronic diseases. In specific, it compared these diseases between vegetarians and non- vegetarians in Jordan in 2012. Questionnaires were distributed and the responses of 97 vegetarians and 97 non-vegetarians were analyzed. Chi-square and Wilcoxon signed ranks tests showed statistically significant differences between the two groups. In particular, chronic diseases including Diabetes, Hypertension, and Obesity were more prevalence among non-vegetarians compared to vegetarian respondents.

## 1. Introduction

Studies which compare disease patterns in people with diverse food lifestyles assist determining factors that cause or avoid diseases. Observational studies demonstrated that vegetarians live longer and were less vulnerable to chronic diseases than non-vegetarians. Burr *et al*. (1981) reported that 85 vegetarians showed on average lower total cholesterol and higher HDL per cents than 214 non-vegetarians. Melby et al. (1994) found that the vegetarians had significantly lower concentrations of serum total cholesterol (STC), LDL-C, triglycerides, STC/HDL-C, and LDL-C/HDL-C than the non-vegetarians. Moreover, they were less hypertensive. Appleby et al. (2009), Ginter (2008) and Brathwaite et al. (2003) found that vegetarians have a lower prevalence of lifestyle diseases such as Obesity, Diabetes and Hypertension than non-vegetarians. Spenser et al. (2003) compared body mass index (BMI) in four diet groups (meat-eaters, fish-eaters, vegetarians and vegans). They reported that age-adjusted mean BMI was significantly different between the four diet groups, being highest in the meat-eaters and lowest in the vegans. Fish-eaters and vegetarians had similar, intermediate mean BMI. Walker et al. (2005) argued that foods rich in animal protein containing higher amounts of fatty acids, are converted to various lipoproteins in the liver leading to increased deposition of adipose tissue, facilitating formation of atheromatous plaques inside the arteries. As a consequence, there is narrowing of the arterial wall leading to increased risk of Hypertension, stroke and coronary arterial diseases. Segasothy and Phillips (1999) studied the positive and adverse effects of vegetarian diets in various medical conditions. They argued that Soybean-protein diet, legumes, nuts and soluble fiber significantly decrease total cholesterol, low-density lipoprotein cholesterol and triglycerides. Diets rich in fiber and complex carbohydrate, and restricted in fat, improve control of blood glucose concentration, lower insulin requirement and aid in weight control in diabetic patients. They also reported an inverse association between nut, fruit, vegetable and fiber consumption, and the risk of coronary heart disease. Craig (2009, 2010) found that vegetarians usually have lower BMI, serum total and low-density lipoprotein cholesterol levels, and blood pressure; reduced rates of death from ischemic heart disease; and decreased incidence of Hypertension, stroke, type 2 Diabetes, and certain cancers than do non-vegetarians. However, vegetarian diets were found to cause deficiencies in vitamin B_12_, vitamin D, ω-3 fatty acids, calcium, iron, and zinc. Consistently, Key et al. (2006) demonstrated that vegetarian diets are usually rich in carbohydrates, n-6 fatty acids, dietary fiber, carotenoids, folic acid, vitamin C, vitamin E and Mg, and relatively low in protein, saturated fat, long-chain n-3 fatty acids, retinol, vitamin B_12_, Zn and Ca. Hoek et al. (2004) investigated socio-demographic characteristics, and attitudes to food and health of vegetarians, non-vegetarian consumers of meat substitutes, and meat consumers in the Netherlands. They found that vegetarians had more positive attitudes towards importance of product information, specialty shops, health, novelty, ecological products, social event, and social relationships than meat consumers. Kennedy et al. (2001) used population representative data from the Continuing Survey of Food Intakes by Individuals (CSFII) to examine the relationship between several health and nutrition indicators and vegetarian diets. In particular, they conducted a cross sectional study on 10,014 subjects aged 19 years and older from the 1994-1996 CSFII in USA. They found that diets that are high in carbohydrate and low to moderate in fat tend to be lower in energy. The lowest energy intakes were observed for those on a vegetarian diet. The diet quality as measured by healthy eating index (HEI) was highest for the high carbohydrate groups and lowest for the low carbohydrate groups. The BMIs were significantly lower for men and women on the high carbohydrate diet; the highest BMIs were noted for those on a low carbohydrate diet. Using questionnaires, Bedford and Barr (2005) provided evidence indicating that vegetarians are more ’health conscious’ than non-vegetarians. In specific, they conducted a cross sectional study for 1817 community-dwelling residents 19–84 years, in British Colombia. Rizzo et al. (2011) documented that vegetarian dietary pattern is associated with a more favorable profile of metabolic risk factors and a lower risk of metabolic syndrome. Key et al. (1999) compared death rates from common diseases of vegetarians with those of non-vegetarians with similar lifestyles. They found that mortality from ischemic heart disease was 24% lower in vegetarians than in non-vegetarians. However, There were no significant differences between vegetarians and non-vegetarians in mortality from cerebrovascular and cancer diseases. Most of the previous studies were in USA and Europe. Studies in other countries such as Japan and China reported that vegetarians have lower serum total cholesterol than the non-vegetarians (Higuchi et al., 2005; Tsuchida, 2007; Sasaki et al., 2004). Similar studies are unique in the Arab countries. This is the first study in Jordan that compares the major chronic diseases between vegetarians and non-vegetarians.

## 2. Method

A questionnaire was used to collect data. It consisted of three parts, the first asked about the demographic characteristics of the respondents, the second asked several yes or no questions to classify the respondents into vegetarians or non-vegetarians and the third part was composed of a number of questions regarding each chronic disease examined, including questions of whether the respondent has this disease or not, how many years, how many in the family. In particular we distributed 400 questioners on people whose ages are approximately over 40 years old of the inhabitants in Ajloun city. We received 286 back. The respondents were divided into vegetarians and non-vegetarians. The number of vegetarian respondents was 97 so that we randomly selected 97 respondents of non-vegetarians for analysis. The validity of the questionnaire was evaluated through expert colleagues in the field. Additionally, we estimated test-retest reliability. In particular, we asked a number of our respondents to fill the same questionnaire after two months and no substantial change in the construct being measured between the two occasions was found. Chi-square and Wilcoxon (1945) signed ranks tests were used to analyze differences between vegetarians and non-vegetarians. Chi-square is a parametric test assuming normal distribution of data while Wilcoxon signed ranks is a non-parametric test. The data were also examined to assess whether demographic differences between vegetarians and non-vegetarians may have influenced the results. The significance level was set at *p* = 0.05 for both statistical measures.

Respondents were categorized by defining vegetarians as subjects who reported consuming meat and poultry >1 time/month. Non-vegetarians were defined as consuming red meat or poultry ≥1 time/month.

## 3. Results and Discussion

A total of 400 questionnaires were distributed. The response rate was 71.5%. 286 questionnaires were received back. Ninety seven respondents were vegetarians. Ninety seven non-vegetarian respondents were randomly selected for comparison.

Table 1 reports the descriptive statistics of the respondents. Approximately half of the respondents were above 60 years old. The males were more than the females among the non-vegetarians. However, the opposite was right for the vegetarians. Some respondents had chronic diseases in their family history, for example 39% (34%) of non-vegetarians (vegetarians) had Hypertension in their family history. Only 11% (18%) of the non-vegetarians (vegetarians) do exercises. Finally, 64% (58%) of the non-vegetarians (vegetarians) were currently smokers. Table 2 reports that 52% of the non-vegetarians had Obesity. On the other hand, 31% of the vegetarians had the same disease. The respondents were asked about their weight and height in order to calculate BMI (BMI=weight in kg/(height in meters)^2^). The respondent were classified as having Obesity when BMI was more than or equal 30.

**Table 1:** Descriptive statistics

		None-Veg	Veg
Age	40-50	20%	15%
	51-60	34%	38%
	Above 60	46%	47%
Gender	male	57%	41%
	female	43%	59%
Smoking		64%	58%
Family History	Obesity	23%	11%
	Diabetes	35%	28%
	Hypertension	39%	34%
Exercise		11%	18%

**Table 2:** Differences in obesity prevalence between vegetarians and non-vegetarians

Obesity		None-Veg	Veg
Yes		52%	31%
No		48%	69%
Chi-Square Statistic	13.228^*^		
Wilcoxon signed ranks test	10.876^*^		

* denotes significant differences at a critical value of 5%. P-values are approximately equal to zero

Table 2 shows statistically significant differences between vegetarians and non-vegetarians according to both Chi-square and Wilcoxon signed ranks tests. Particularly, vegetarians had less cases of Obesity than non-vegetarians.

Table 3 reports that Hypertension was more prevalence among non-vegetarians compared to vegetarian respondents. The difference is statistically significant according to both Chi-square and Wilcoxon signed ranks tests. Specifically, 63% of the non-vegetarians had Hypertension while 51% of vegetarians had the same disease. Hypertension was found to be the most common disease among the respondents regardless whether they were vegetarians or non-vegetarians.

**Table 3:** Differences in hypertension prevalence between vegetarians and non-vegetarians

Hypertension		None-Veg	Veg
Yes		63%	51%
No		37%	49%
Chi-Square Statistic	15.987^*^		
Wilcoxon signed ranks test	11.968^*^		

* denotes significant differences at a critical value of 5%. P-values are approximately equal to zero

Consistent with the previous results, Table 4 shows that Diabetes was significantly more prevalence among non-vegetarians compared to vegetarian respondents. 44% (38%) of the non-vegetarians (vegetarians) had Diabetes.

**Table 4:** Differences in diabetes prevalence between vegetarians and non-vegetarians

Diabetes		None-Veg	Veg
Yes		44%	38%
No		46%	72%
Chi-Square Statistic	11.765^*^		
Wilcoxon signed ranks test	10.342^*^		

* denotes significant differences at a critical value of 5%. P-values are approximately equal to zero

A recent study for the world health organization (WHO) (2010) considered Jordan as one of the top ten countries with the highest rates of Diabetes and heart diseases. A survey conducted by the ministry of health in 2009 showed that more than one million and 200 thousand Jordanians (approximately 20% of the population) suffer from Diabetes and Hypertension. Lifestyle food is certainly a central cause of these diseases. This cross-sectional survey indicated that a vegetarian lifestyle food exerts an effect in decreasing the prevalence of chronic diseases. There was an overall lower prevalence of Diabetes, Hypertension, Obesity among vegetarians compared to non-vegetarians. The differences between the two groups were statistically significant according to both parametric and non-parametric tests. Vegetarian foods are characterized by a reduced amount of fat, and no meat or animal fat. Indeed, some studies showed protein-rich and fat-rich foods of animal origin are associated with a higher prevalence of chronic diseases. Recently, Rizzo et al. (2011) reported that triglycerides, glucose, blood pressure levels, waist circumference, and BMI were significantly lower (P≤0.05) in vegetarians than in non-vegetarians. Appleby et al. (2009) and Brathwaite et al. (2003) found consistent results.

## 4. Conclusion

This study investigated the effect of lifestyle food on chronic diseases in Jordan. Surveys were used to compare between people who do not eat red meat and poultry (vegetarians) and who do (non-vegetarians). The answers of 194 respondents indicated lower prevalence of Diabetes, Obesity, and Hypertension among vegetarians than non-vegetarians. Both parametric and non-parametric tests showed statistically significant differences between the two groups. Overall, vegetarian food lifestyle is recommended to decrease the probability of chronic diseases for healthy people.
